# A Case of ST-Elevation Myocardial Infarction With Right Bundle Branch Block, an Ominous Sign of Critical Coronary Occlusion

**DOI:** 10.7759/cureus.21216

**Published:** 2022-01-13

**Authors:** Hajira Basit, Alexa Kahn, Seyed Zaidi, Hal Chadow, Abdullah Khan

**Affiliations:** 1 Internal Medicine, Brookdale University Hospital Medical Center, Brooklyn, USA; 2 Cardiology, State University of New York Downstate Medical Center, Brooklyn, USA; 3 Cardiology, Brookdale University Hospital Medical Center, Brooklyn, USA

**Keywords:** cardiogenic shock, primary percutaneous coronary intervention (pci), right bundle branch block, left anterior descending stenosis, st-elevation myocardial infarction (stemi)

## Abstract

Coronary artery disease is one of the leading causes of death worldwide, and ST-elevation myocardial infarction (STEMI) is one of its most serious manifestations. While STEMI itself is an ominous sign, there are other sinister electrocardiogram (EKG) patterns that are associated with increased morbidity and mortality, one of which is STEMI with right bundle branch block (RBBB). Blood supply to the right bundle comes from the left coronary circulation. Intuitively, RBBB in the setting of anterior wall myocardial infarction would indicate more extensive myocardial involvement and thus portend a worse prognosis. This case presents the significance of the association of new RBBB with critical lesions of the left anterior descending artery (LAD), therefore a low threshold for emergent coronary angiography and percutaneous coronary intervention (PCI).

A 63-year-old man with a known history of non-insulin-dependent diabetes mellitus (NIDDM), hypertension, and hypertriglyceridemia non-compliant with medications presented to the emergency department (ED) after a visit with his primary care physician, with a chief complaint of exertional substernal chest pain for a one-week duration. His EKG on arrival showed significant ST-segment elevation with an atypical EKG pattern showing RBBB in V1-V2 with ST depression in reciprocal leads. Cardiac biomarkers showed an initial troponin I value of 0.441 ng/mL. Due to his persistent, worsening chest pain and associated nausea with episodes of vomiting, he was taken for an emergent cardiac catheterization that revealed a 100% lesion in his proximal LAD. The procedure was complicated by the development of cardiogenic shock requiring intra-aortic balloon pumps and vasopressors. A successful primary PCI was performed with drug-eluting stent (DES) to the 100% lesion in the proximal LAD and DES to the 80% lesions in the mid LAD, with 0% residual stenosis after the intervention. There was thrombolysis in myocardial infarction (TIMI) 0 flow pre-procedure and TIMI 3 flow post-intervention. Left ventriculography revealed anterolateral akinesis, apical akinesis, and diaphragmatic hypokinesis with an estimated ejection fraction (EF) of 20%. Transthoracic echocardiogram was repeated prior to discharge. Left ventricular (LV) systolic function was normal by visual assessment, and EF was noted to be ~55%. The patient continued on dual antiplatelet therapy and the rest of goal-directed medical therapy for coronary artery disease post-procedure.

New-onset RBBB in the patient with typical STEMI in the context of ischemic symptoms should raise suspicion of critical proximal LAD coronary occlusion. It is increasingly being recognized as one of the significant EKG patterns for occlusive myocardial infarction associated with the worst outcome and mortality, highlighting the need to pay critical attention to these patients. Given the poor prognosis of these patients in the setting of acute myocardial infarction (AMI), it is essential to minimize the delay in initiating reperfusion therapy as they can potentially benefit from emergent intervention.

## Introduction

Coronary artery disease is associated with increased morbidity and is one of the leading causes of death worldwide [1]. One of the most serious manifestations of coronary artery disease is ST-elevation myocardial infarction (STEMI). Typically, STEMI in everyday practice is an indication for urgent reperfusion therapy in the presence of other stigmata of acute coronary syndrome (ACS) events [2]. While STEMI itself is an ominous sign, there are further sinister electrocardiogram (EKG) patterns that are associated with increased morbidity and mortality, one of which is STEMI with right bundle branch block (RBBB). Blood supply to the right bundle comes mostly from septal branches of the left anterior descending coronary artery, a branch of the left coronary circulation, which also supplies other major dominant areas of the myocardium. Intuitively, RBBB in the setting of anterior wall myocardial infarction would indicate more extensive myocardial involvement and thus would portend a worse prognosis. Here, we present a case of a new-onset RBBB with chest pain found to have critical lesions of the left anterior descending artery (LAD) complicated by the development of cardiogenic shock intra-procedure.

## Case presentation

**Figure 1:**
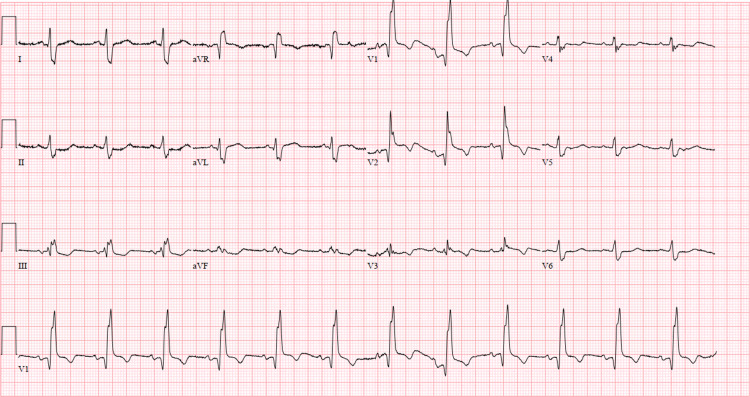
EKG on arrival EKG, Electrocardiogram.

A 63-year-old man with a known history of non-insulin-dependent diabetes mellitus (NIDDM), hypertension, and hypertriglyceridemia who was non-compliant with medications presented to our institution after visiting his primary care physician with a chief complaint of exertional substernal chest pain for a one-week duration. On the day of the presentation, he was given aspirin in the ambulatory clinic due to worsening symptoms, which did not alleviate his pain. He had no known cardiac history. His EKG on arrival revealed a new RBBB with significant ST-segment elevation in leads V1-V2 (Figure 1).

**Figure 2:**
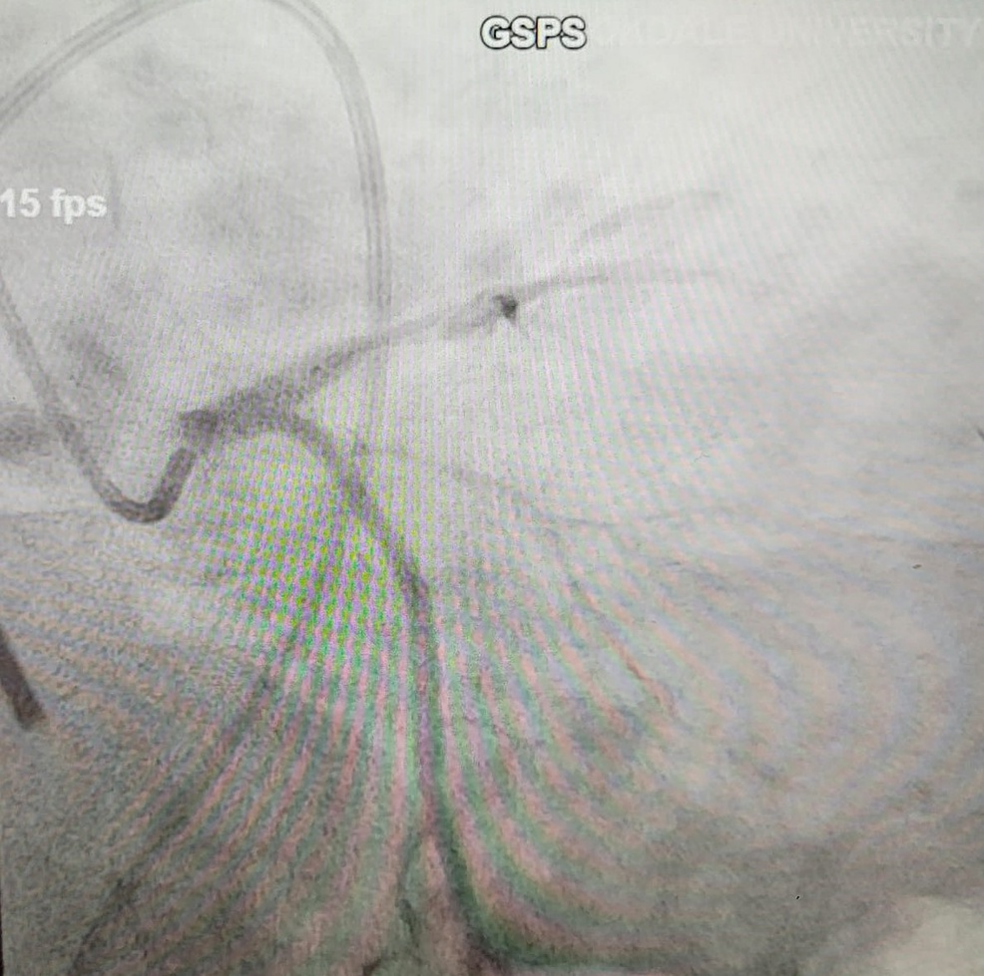
Cath image showing critical stenosis of proximal LAD LAD, Left anterior descending artery.

Cardiac biomarkers showed an initial troponin I value of 0.441 ng/mL. Due to his persistent, worsening chest pain and associated nausea with episodes of vomiting, he was taken emergently for cardiac catheterization that revealed a 100% lesion in his proximal LAD (Figure 2). 

**Figure 3:**
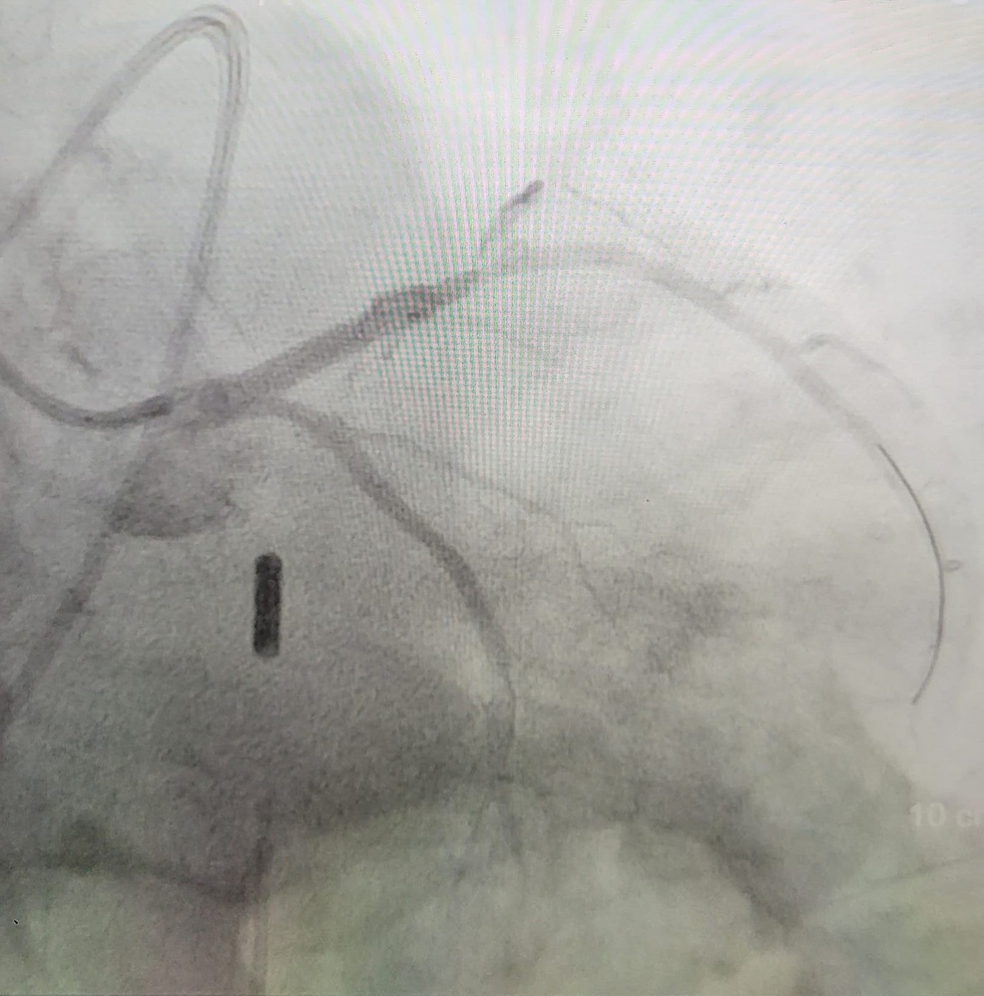
Cath image post-intervention

His course was complicated by the development of cardiogenic shock requiring the insertion of an intra-aortic balloon pump (IABP) and vasopressor support. A successful primary PCI was performed with a drug-eluting stent (DES) of the 100% lesion in the proximal LAD and 80% lesions in the mid LAD with a 0% residual stenosis. There was thrombolysis in myocardial infarction (TIMI) 0 flow pre-procedure and TIMI 3 flow post-intervention (Figure 3).

Left ventriculography revealed anterolateral akinesis, apical akinesis, and diaphragmatic hypokinesis with an estimated ejection fraction (EF) of 20%. Transthoracic echocardiogram was repeated prior to discharge. LV systolic function was normal by visual assessment with an EF of 55%. The patient continued with dual antiplatelet therapy and guideline-directed medical therapy for acute myocardial infarction (AMI) disease post-procedure. The IABP was removed on hospital day 2.

## Discussion

Myocardial infarction remains a leading cause of mortality worldwide despite all of the recent advancements in medical management and device therapy. The most common cause is acute vessel closure secondary to plaque rupture causing prolonged ischemia, which results in myocardial cell death evidenced by an increase in cardiac enzymes, specifically troponin. Arriving at a diagnosis of MI requires integration of clinical findings, patterns on the ECG, laboratory data, observations from imaging procedures, and on occasion pathological findings.

Although STEMI accounts for 38% of all myocardial infarction in the United States, the STEMI mortality rates supersede the non-STEMI (NSTEMI) rates [3]. STEMI is an urgent indication for reperfusion therapy either via percutaneous intervention or thrombolysis. However, there are special considerations where the presence of additional EKG patterns may portend a worse prognosis. A new RBBB in the presence of ST elevation in the anterior precordial leads is one such pattern and indicates a critical proximal occlusion of the LAD, therefore demands urgent attention as the blood supply of the right bundle branch is primarily from the LAD [4].

The case presented here highlights the association between a new RBBB in STEMI and AMI. A meta-analysis by Hazem et al. to investigate the relationship between AMI and new or old RBBB found a greater all-cause 30-day mortality compared to those without a bundle branch block [5]. Similarly, a retrospective analysis compared patients with a primary diagnosis of anterior wall myocardial infarction (AWMI) with an RBBB to patients with an AWMI and no RBBB, and it was found that the presence of RBBB was a significant independent predictor of poor prognosis, including a higher rate of acute heart failure, complete heart block, and the need for a permanent pacemaker, as well as higher in-hospital mortality [6].

Wang et al. also evaluated the prognostic value of a new RBBB with proximal LAD occlusion and found that a new RBBB is likely to have a higher incidence of cardiogenic shock and increased long-term mortality [4]. In these conditions, urgent reperfusion has been shown to significantly improve the outcomes. In light of the multiple studies suggesting the association between RBBB and AMI, the latest European Society of Cardiology guidelines for AMI management now recommend a primary PCI when patients with RBBB show persistent ischemic symptoms [7]. All of the above studies further strengthen the association between RBBB and AMI where urgent reperfusion therapy is associated with a significant improvement and favorable outcomes.

## Conclusions

New-onset RBBB in a patient with typical STEMI in the context of ischemic symptoms should raise suspicion of critical proximal LAD coronary occlusion. It is increasingly being recognized as one of the significant EKG patterns for occlusive myocardial infarction associated with the worst outcome and mortality, highlighting the need to pay critical attention to these patients. Given the poor prognosis of these patients in the setting of an AMI, it is essential to minimize the delay in initiating reperfusion therapy as they can potentially benefit from the emergent intervention.
